# Priors and prejudice: hierarchical predictive processing in intergroup perception

**DOI:** 10.3389/fpsyg.2024.1386370

**Published:** 2024-06-13

**Authors:** H. T. McGovern, Marte Otten

**Affiliations:** ^1^School of Psychology, The University of Queensland, Brisbane, QLD, Australia; ^2^Brain & Cognition Group, Department of Psychology, University of Amsterdam, Amsterdam, Netherlands

**Keywords:** prejudice, Bayesian, intergroup, bias, social, cognition, racism

## Abstract

Hierarchical predictive processing provides a framework outlining how prior expectations shape perception and cognition. Here, we highlight hierarchical predictive processing as a framework for explaining how social context and group-based social knowledge can directly shape intergroup perception. More specifically, we argue that hierarchical predictive processing confers a uniquely valuable toolset to explain extant findings and generate novel hypotheses for intergroup perception. We first provide an overview of hierarchical predictive processing, specifying its primary theoretical assumptions. We then review evidence showing how prior knowledge influences intergroup perception. Next, we outline how hierarchical predictive processing can account well for findings in the intergroup perception literature. We then underscore the theoretical strengths of hierarchical predictive processing compared to other frameworks in this space. We finish by outlining future directions and laying out hypotheses that test the implications of hierarchical predictive processing for intergroup perception and intergroup cognition more broadly. Taken together, hierarchical predictive processing provides explanatory value and capacity for novel hypothesis generation for intergroup perception.

## Introduction

Insights from neuroscientific research have improved understanding of how intergroup bias is formed, strengthened, and maintained (Cikara and Van Bavel, 2014; Bagnis et al., 2019; McGovern and Vanman, 2020; Amodio and Cikara, 2021 for reviews). At the same time, emergent literature suggests computational accounts of social cognition could explain how intergroup attitudes are encoded and how they impact behavior (Freeman and Ambady, 2011; Xiao et al., 2016; Hackel and Amodio, 2018; Amodio, 2019). Computational frameworks provide insight into how prejudice or stereotypes can shape early cognitive processing, such as intergroup perception. One influential framework combining knowledge from neuroscience and computational approaches is hierarchical predictive processing (from here on predictive processing (Friston, 2010; Hohwy, 2013). Here, we suggest that predictive processing provides a cohesive account of biased intergroup perception. This framework can offer a degree of theoretical parsimony not offered by competing frameworks and enables novel hypothesis generation (see Table 1). Adoption of this framework, in turn, may enable an improved understanding of biased intergroup perception, offering space for the creation of novel interventions for reducing intergroup bias.

**Table 1:** Outline of possible experimental setups and predictions arising from a predictive processing framework.

Experiment/Paradigm	Prediction
Drift diffusion modeling	Higher starting point (i.e., prejudice) should be accompanied by higher drift rate toward the prejudiced prior. This would be evidence for the idea that agents preferentially seek sensory data consistent with priors.
Perceptual decision making	The presentation of a stereotyped context or object should push identification toward stereotype-consistent objects/faces when visually ambiguous object is subsequently presented. For example, after being in a threatening context, participants could be more likely to identify a racially ambiguous face as a member of a stereotyped group—particularly the case for most prejudiced participants. This could also work the other way where the presentation of a stereotyped group member should mean subsequent presentation of a stereotype-consistent context is more likely to be associated with the stereotyped group. Another way of measuring this could be reaction times—threatening stimuli could be faster to enter awareness after observing stereotyped group members, and vice versa.
Classical conditioning	Conditioning stimulus (e.g., frightening stimulus) could be paired with an unconditioned stimulus (e.g., in group and outgroup member) with varying probabilities in the conditioning phase. Subsequent presentation of an unconditioned stimulus should cause increased fear expression, and thus the psychophysiological alterations could vary as a function of prior probabilities of a conditioned stimulus with an unconditioned stimulus. Moreover, prejudiced individuals should be slower to extinguish in the extinction phase.
In attentional blindness	Following the presentation of a stereotype-consistent stimulus (e.g., anger in a Black face or threat-connoting context), participants should be more likely to notice members of stereotyped members of outgroups, when subsequently presented with an emotionally neutral scene (e.g., seeing a Black face). This would be evidence of tuned attention toward stereotyped priors following the introduction of motivational or perceptual cues. This could also work the other way where the introduction of a stereotyped group member (e.g., Black face) may increase the probability of noticing a fear-connoting stimulus during a subsequent IB task.
Testing prediction errors	Largest prediction errors (e.g., represented by the N170 signal) could vary as a function of attention toward stereotype-inconsistent information. For example, researchers could pair stereotype-consistent stimulus with a member of a stereotyped group, unexpected pairing of the US with a member of a non-stereotyped group, and pairing of a stereotyped group with a stereotype-inconsistent US. The latter two pairings should solicit a prediction error as measured by an P100 or N170 signal. The amplitude of this response should also increase as a function of the strength of the departure from initial expectations (i.e., how likely did the participant deem the US to be paired with the CS).

## Hierarchical predictive processing

Bayes theorem suggests that in estimating the probability of any event, one should consider the current evidence for that event but also the prior knowledge about the event. Predictive processing is a theory that translates this insight to cognition, specifically focusing on perception (Joyce, 2019). In this framework, a perceiver has an internal “model” of the outside world and continuously updates this model based on new information (Kersten et al., 2004; Knill and Pouget, 2004; Bar, 2007; Friston and Kiebel, 2009; Friston, 2010; Clark, 2013; Hohwy, 2013, 2017; Otten et al., 2017; Williams, 2018).

The predictive processing framework assumes that any perceptual process, where the organism acquires new information about the outside world in the form of sensory information, results in an inference about the cause of that sensory data, a so-called *posterior*. Each *posterior* is a combination of the *likelihood* and *prior*. The *likelihood* is a representation of the data sampled in the sensorium, which includes both the sensory input and an estimate of the reliability of that sensory data. The *prior* is the initial prediction (before any sensory information is encountered) about the cause of the sensory data. The prior is often shaped by memory and thus by previous experiences.

In hierarchical predictive processing—namely, active inference under deep generative models—there are priors at every level of the implicit model. Prior beliefs at intermediate levels are known as ‘empirical’ priors because they are updated by evidence from lower levels. In short, empirical prior beliefs depend upon experience. This is often reflected in the description of belief updating, which uses today’s posteriors as tomorrow’s priors.

Priors can be held with greater or lesser precision. A very precise conviction or commitment to a prior belief (e.g., innate subpersonal priors that underwrite homeostasis) is clearly less amenable to updating. Conversely, prior beliefs at higher levels are generally held with less precision or confidence and are more amenable to updating, through perceptual inference, or long-term revision, through learning and experience-dependent plasticity.

The predictive processing framework has been successfully applied to understanding visual perception, supported by a wealth of research in recent years, suggesting that it offers a plausible account for how visual information is encoded, represented, and predicted in cognitive systems (Mumford, 1992; Rao and Ballard, 1999; Spratling, 2010, 2017; Rauss et al., 2011; Bastos et al., 2012; Clark, 2013; Kok and de Lange, 2015; Hindy et al., 2016; Schellekens et al., 2016; Shipp, 2016; Edwards et al., 2017; Homann et al., 2017; Alexander and Brown, 2018; Friston, 2018; Hogendoorn and Burkitt, 2018; Keller and Mrsic-Flogel, 2018; Pereira et al., 2019). It is worth noting a recent review that found mixed support for key tenets of predictive processing (Walsh et al., 2020). In particular, the review found partial support that the process of minimizing prediction error occurs via an inferential hierarchy (see below), moderate support for the notion that expectations attenuate sensory processing, and modest support for the notion that error signals should be larger insofar as sensory data departs from expectation. For what follows, it should thus be noted that predictive processing still has several empirical hurdles to clear to be considered a comprehensive approach to brain function, and future work remains to test and refine key tenets of predictive processing. Acknowledging these limitations, we nonetheless move forward with the assumption that predictive processing at least offers a useful framework for conceptualizing brain functioning and perception.

Predictive processing departs from classical notions of the brain as a passive perceiver of information, recasting perception as the brain’s “best guess” of the hidden causes of data in the sensorium (see Friston, 2010). Predictive processing assumes that the trade-off between priors, likelihood, and posteriors is all done in the service of minimizing prediction error—the divergence between expected and sampled sensory data. In this process, the brain generates a model of the world in the service of prediction error minimization. The modeling of the world is thus generative in the sense that it optimizes the inferred causes of sensory signals based on experience and internally generates a set of predictions (priors) about what kinds of sensory encounters it is likely to sample thereafter. Hierarchical predictive processing (or coding) is also known as active inference and is distinguished from predictive coding accounts of perceptual synthesis by emphasizing the enactive and situated aspect of sense-making (e.g., active vision or, more generally, active inference that involves active sampling of the sensorium).

Predictive processing has been applied to understand a range of cognitive processes, from psychiatric disorders (Corlett et al., 2019; McGovern et al., 2022) to emotion perception (Ransom et al., 2020). A growing body of research suggests that perceptions (even if incorrect) depend upon prior learning (Gilbert et al., 2001; Knill, 2007; Powers et al., 2017) and local context (Palmer, 1975; Bar, 2004, 2007; Snyder et al., 2015 for an overview). For example, seeing lips move a certain way makes people likely to hear a word even if there is no actual audio input (Haarsma et al., 2020). Similarly, hearing a tone alongside a visual stimulus makes people more likely to report hearing the tone alongside the visual stimulus even if it no longer remained (Powers et al., 2017). Samaha et al. (2018) found that verbal cues increased recognition and discrimination of ambiguous sensory data. Moreover, improved discrimination was predicted by increased power of the posterior alpha band, suggesting that the previously learned knowledge tuned the brain for future perception (via modulation of alpha-band oscillations), which attenuated early visual attention and thus perception (Samaha et al., 2018, pp. 1; see also Mayer et al., 2015; Zhou et al., 2021). This rests upon electrophysiological evidence that ascending prediction errors are conveyed by fast gamma activity while descending predictions are mediated at slower frequencies (Bastos et al., 2015; Michalareas et al., 2016; Pefkou et al., 2017). These spectral asymmetries are just one facet of the empirical evidence for predictive coding in the brain that complements other anatomical and physiological studies (please see Shipp, 2016; Hohwy, 2020; Haarsma et al., 2022; Hodson et al., 2024 for review).

This leaves us two important implications for what follows. First, prior expectations can have a large effect on perceptual processing (Knill and Pouget, 2004; Friston, 2010; Hohwy, 2013, 2017). Perhaps most importantly, expectations (i.e., priors) can result in perceptions not afforded by the actual sensory data, meaning people can see or hear things that are not actually present.

## Hierarchical predictive processing in intergroup perception: review of evidence

In what follows, we outline evidence of how priors can influence social and intergroup perception. We assess three lines of evidence. First, we assess the influence of group-based priors on face perception. Second, we assess how group-based priors influence emotional perception. Third, we assess how group-based priors may influence object perception. In synthesizing these complementary lines of evidence, we aim to illustrate the tangible influences that group-based priors can enact on the perpetual process. Note the intention at this point is not to say that this evidence points only to hierarchical predictive processing (this is discussed in the section following the evidence review where we argue how predictive processing may offer the most parsimonious explanations of these findings), before offering predictions and questions for future work (see Table 1 for summary).

### Social priors and face perception: early and late processing

Social knowledge about others can modulate early visual perception (Galli et al., 2006; Anderson et al., 2011; Otten et al., 2017 for review). Social knowledge can be based on explicit information about other individuals, such as group membership (Anderson et al., 2011). Stereotypes and prejudices held toward other groups can alter even the earliest phases of intergroup perception (Golby et al., 2001; Hugenberg and Bodenhausen, 2003; Freeman et al., 2011; Freeman and Ambady, 2011; Van Bavel et al., 2011; Ratner and Amodio, 2013; Xiao et al., 2016; Villiger, 2023). In one example, Levin and Banaji (2006) showed that when a face with Black features or White features was shown with identical luminance, participants reported the face with the Black features as darker than the White face. This suggests that the features of the face activated priors about skin color, which, in the absence of conclusive sensory data, influenced the posterior percept representing skin color (see Firestone and Scholl, 2015).

Further evidence of the constraining effect of priors comes from evidence assessing face perception (Trapp et al., 2018; Pereira et al., 2019; Walsh et al., 2020 for an overview). Neural signals involved in early processing relate to the visual processing of faces, such as the N170. The N170 is a neuronal signal with a negative peak occurring over the occipital lobe around 170 ms following stimulus onset (Rossion and Jacques, 2012; Hinojosa et al., 2015). This component is increasingly thought to reflect prediction errors, such that an increased amplitude corresponds to an unexpected stimulus in the visual field (Johnston et al., 2016, 2017; Robinson et al., 2020; Baker et al., 2022). Johnston et al. (2016) had participants belonging to a more frequently presented identity, a less frequently presented identity, and a random identity. Low frequency and randomly presented identities induced an N170 response over the occipital lobe. Johnston et al. (2016) suggest that the visual system detects mismatches (here between the expected and actual identity of a face or prediction errors) between expected and sampled visual data, in line with predictive processing.

The N170 component is implicated in social (Johnston et al., 2016; Walla et al., 2020) and even intergroup perception (Ito et al., 2004; Galli et al., 2006; Bentin et al., 2007; Ibáñez et al., 2010). If the N170 component indeed (at least partially) represents prediction error, then stereotype-inconsistent face information should solicit an N170 response. An extensive literature lends support to this notion (Ito and Urland, 2003; Herrmann et al., 2007; Stahl et al., 2008; Ibáñez et al., 2010; Ofan et al., 2011). Ibáñez et al. (2010), for example, found that outgroup faces produced an increased N170 response when paired with positive words but not negative words. The fact that the brain detects a departure between expectations and sampled data in the sensorium (a.k.a. prediction error) this early in the visual process lends support to the idea that group-based priors can shape even the earliest stages of visual perception.

### Group stereotypes and emotion perception

It is not only person-level knowledge that influences early perception. Social knowledge about groups, such as stereotypes, modulates how individuals from that stereotyped group are subsequently perceived and acted toward (Payne, 2001; Correll et al., 2002; Eberhardt et al., 2004; Unkelbach et al., 2008; Villiger, 2023). The effect of social knowledge on intergroup perception occurs across multiple processes, including quicker detection of anger in ambiguous facial expressions for Black compared to White faces (Hugenberg and Bodenhausen, 2003; Hutchings and Haddock, 2008), and differential mental representations of emotions displayed by Black and White faces (Otten and Banaji, 2012). This is in line with the stereotype-based association between Black men and aggression (Hugenberg, 2005; Ackerman et al., 2006; Hutchings and Haddock, 2008; Kang and Chasteen, 2009; Shapiro et al., 2009; Zebrowitz et al., 2010; Neuberg and Schaller, 2016; Kleider-Offutt et al., 2018; Thiem et al., 2019; Raissi and Steele, 2021). Stolier and Freeman (2016a) found that brain areas involved in social category representations, the right fusiform gyrus, and the occipital frontal lobe were activated most when viewing Black and Male faces, both of which are linked to the “dangerous” stereotype (also see Stolier and Freeman, 2016b). The authors suggested this was due to the fact these groups share similar stereotypes, impacting early perceptual processes and neural activation.

Related literature shows an increased probability of misperceiving anger in Black compared to White faces (Hugenberg, 2005; Ackerman et al., 2006; Hutchings and Haddock, 2008; Kang and Chasteen, 2009; Shapiro et al., 2009; Zebrowitz et al., 2010; Kleider-Offutt et al., 2018; Thiem et al., 2019; Raissi and Steele, 2021) particularly for those higher in safety concern (Cook et al., 2018) and implicit prejudice (Hutchings and Haddock, 2008). Those with higher safety concerns and self-protection motivation are also more likely to perceive anger in Arabian versus White faces (Becker et al., 2011).

The link between stereotypes and emotion perception is not limited to racial or ethnic stereotypes. Perceived gender also impacts emotion perception (Craig and Lee, 2020, for review). Female faces are faster to be perceived as happy (Becker et al., 2007; Aguado et al., 2009; Tipples, 2019), whereas male faces are faster to be perceived as angry (Le Gal and Bruce, 2002; Smith et al., 2017; Taylor, 2017; Primbs et al., 2022). Craig and Lee (2020) suggest that the tendency to see happiness in female faces is linked to the (implicit) association between female and positive words (Bijlstra et al., 2010; Craig and Lipp, 2018). Similarly, the effects of stereotypes on emotion perception are not limited to facial expressions; the perception of posture is also influenced. Faces are not the only source of social information. Karmali and Kawakami (2023) found that Black targets with an expansive body posture were perceived as more aggressive than White targets with the same expansive body posture. Taken together, these findings suggest that (higher-order) group-level knowledge, such as racial or sex stereotypes, bears influence on subsequent emotion perception.

### Group stereotypes influence the perception of objects and bodies

Group-based social priors do not just alter the perception of faces or the perception of emotion in those faces; there is also evidence that object perception is altered. For example, when shown images of a Black or White face, followed by an image of a weapon or a tool, people are quicker to and more often correctly identify targets as weapons after Black faces are presented compared to when White faces are presented, and even misperceive tools as weapons (Eberhardt et al., 2004; Payne et al., 2005; Payne, 2006; Kidder et al., 2018). These effects of race primes on object perception extend beyond the categorization of the object itself (gun vs. not a gun); they are also visible in decisions to shoot or not shoot in a first-person shooter game based on the presumed presence of a gun (Correll et al., 2002, 2007; Plant and Peruche, 2005; Sadler et al., 2012; Mange et al., 2015). In these experiments, participants should only shoot when the person on the screen carries a gun and not shoot when the person is carrying a different object, such as a phone. These decisions to shoot differ depending on the race of the person that is on screen: participants are faster to shoot a Black gun-carrying target (compared to a White gun-carrying target) while they are slower to not shoot a Black unarmed target compared to White unarmed target (see Mekawi and Bresin, 2015 for a meta-analysis), particularly in participants who are led to associate Black faces with danger (Correll et al., 2002).

Together with the studies on race-priming on gun identification, these studies show that group-based priors can shape how people perceive and respond to objects and that this can confer detrimental real-world consequences. Of course, this work is not as conclusive on whether top-down knowledge would exert its effects in the absence of sensory cues. Future work should continue to disambiguate whether top-down knowledge exerts effects on weapon awareness (i.e., whether we are faster to be aware of weapons when primed with a Black but not White face; see Stein et al., 2023) or whether the effect of race only matters after initial perceptual processing (Freeman and Ambady, 2011; Firestone and Scholl, 2015).

## Advantages of predictive processing

Having discussed evidence of the effect of top-down knowledge on intergroup perception, we now turn to the discussion of the advantages of conceptualizing such findings through a predictive processing lens. We start by comparing predictive processing to alternative theories of social perception, including grounded cognition (Barsalou, 2008) and the dynamic interactive theory of person construal (DIPT) (Freeman and Ambady, 2011). We then outline the theoretical advantages conferred by predictive processing before outlining hypotheses and directions for future work.

### Hierarchical predictive processing compared to other models of social perception

Work dating back to the 1980s (McCauley et al., 1980) has utilized the Bayesian approach to understanding intergroup perception (Hinton, 2017; Otten et al., 2017; Villiger, 2023), social cognition (Freeman and Ambady, 2011; Shafto et al., 2012; Gershman and Cikara, 2020; Vélez and Gweon, 2021), and impression formation (Kunda and Thagard, 1996; Van Rooy et al., 2003; Willis and Todorov, 2006). The challenge remaining here, then, is to explain why predictive processing, as a particular implementation of the Bayesian Brain Hypothesis, offers explanatory and hypotheses-generating advantages.

Predictive processing departs from alternate frameworks in several ways. Alternate frameworks often assume that social knowledge comes online only after the percept reaches awareness (Barsalou, 2008). Grounded cognition, for example, posits that social cognition is an internal simulation of re-enactment and implements shared knowledge from various modalities during the perceptual process (Barsalou, 2008). Contrary to predictive processing, grounded cognition posits that these internal simulations are only influential *following* the initial processing of sensory input of some target stimulus (Barsalou, 2003, 2008; Decety and Grèzes, 2006; Goldman, 2006; Meyer and Damasio, 2009). A shortcoming of this framework becomes apparent when considering binocular rivalry findings. These experiments show effects of motivation (Balcetis and Dunning, 2006; Balcetis et al., 2012; Radel and Clément-Guillotin, 2012), affective state (Jolij and Meurs, 2011), and expectation (Lupyan and Ward, 2013; Pinto et al., 2015; Seth, 2015) on the eventual percept. These internally generated priors not only influenced what participants were likely to report but also whether the stimulus reached consciousness at all. If it were the case that internal models are important only following perception, as suggested by grounded cognition, it remains unclear how internal expectations can influence what is perceived before the perceiver is even conscious of the percept. Predictive processing, on the other hand, posits that percepts arise from internally generated predictions rather than a faithful read out of sense data (Seth, 2013; Barrett and Simmons, 2015). Resultingly, expectations based on experience play a crucial role in what information is searched for and is preferred in visual search space. In turn, predictive processing suggests that no initial stimulus needs to be there to generate a percept in line with these expectations. This would explain why predictions can be generated in the absence of conscious awareness and do not require sensory stimuli. Indeed, the phenomena of dreaming—during rapid eye movement sleep—speaks of the fact that the brain can generate percepts while sequestered from the sensorium [please see (Hobson, 2009) for treatment of this under predictive processing].

Another influential theory that has arisen in recent years is Dynamic Interactive Theory of Personal Construal (from here on DIPT). DIPT builds on ideas from connectionism (Read and Miller, 1993; Kunda and Thagard, 1996; Read et al., 1997; Smith and DeCoster, 1998, 1999; Zebrowitz et al., 2003; Van Overwalle, 2007; Van Overwalle and Labiouse, 2013) and dynamical systems theory (Rumelhart et al., 1986; Smolensky, 1990; Van Gelder and Port, 1995; Rogers and McClelland, 2004). In essence, DIPT assumes that perception of others is gradually built up via cyclical interactions between “categories, high-level cognitive states, and the low-level processing of facial, vocal, and bodily cues” (Freeman and Ambady, 2011, pp. 250). Once a perceptual cue is presented, it begins a cascade of interaction between neural nodes of mutual inhibition and excitation that encode social categories (i.e., if there is a male visual cue, then this might excite the angry category and inhibit the happy category), the net process of which is a percept that integrates bottom-up social input and top-down social knowledge (Freeman and Ambady, 2011). However, this is also difficult to reconcile with research that purposely exposes participants to ambiguous stimuli. In such cases, participants frequently rely on existing priors and can arrive at a percept without clear sensory evidence belonging to a clear category (Tversky and Kahneman, 1974; Correll et al., 2002, 2014; Eberhardt et al., 2004; Neth and Gigerenzer, 2015). The fact that perceptual inferences and judgments can be determined without categorical cues offers support to a predictive processing framework, given the fact that the cognitive system (in this circumstance) can generate a percept without a clear stimulus to kickstart the bottom-up/top-down integration proposed by DIPT. Predictive processing suggests that when there is ambiguous sensory data at hand, the brain reverts to priors in attempting to generate predictions about what is causing the sensory data. We would thus expect that when there are unclear perceptual cues (i.e., sensory data that does not clearly belong to any particular category), resulting percepts would be most likely to be consistent with the social agents prior (i.e., existing expectations based on past experience). More precisely, if (under DIPT) there is highly ambiguous data at hand, it does not offer an explanation of how the brain then makes inference on what category the sensory data belongs to (e.g., whether a conspecific whom we cannot see well belongs to one race over another). In this situation, predictive processing suggests that the category (e.g., what racial category does this person belong to) under ambiguous circumstances can be arrived at purely based on prior experience. As such, prior experience tilts the perceptual judgement arrived at toward that consistent with prior experience.

Perhaps a key departure of predictive processing (and why it carries explanatory advantage) is the emphasis placed on attention (Friston, 2010). DIPT outlines that once a percept reaches awareness, it begins a cascading sequence of interactions between excitatory/inhibitory nodes (Freeman and Ambady, 2011), which enables a convergence toward an eventual social percept. However, predictive processing suggests that agentic motives, the social environment, and social knowledge tune the types of information being attended to or sought out in the first instance (Hung, 2023). This is particularly important in explaining how ambiguous stimuli are more likely to be perceived in line with preexisting stereotypes (Hugenberg and Bodenhausen, 2003; Rahman and Sommer, 2012).

### Why is prejudice hard to undo? Attention, precision, and prediction errors

As discussed above, environmental cues can influence what percepts form during social perception, and agents’ stronger priors can be uniquely difficult to undo. This is a particular problem for prejudice, as the most strongly prejudiced beliefs could be the most difficult to undo (Hung, 2023). Indeed, more strongly held beliefs are less amendable to update than those less strongly held (Kunda and Sinclair, 1999; Sinclair and Kunda, 1999; Jost et al., 2017), decreasing the probability of belief update, independent of their accuracy (Kunda and Sinclair, 1999; Sinclair and Kunda, 1999). Recent research underscores how motivation can influence early perceptual and subsequent categorization processes—deemed motivated perception (Leong et al., 2019). Participants were instructed to complete a task of visual categorization. Each participant was presented with a composite image of a face within a visual scene, and participants were instructed to say whether the scene consisted of more face, or more background scene. Subsequently, participants were told that they could win money if the next image was of a particular category (i.e., face or scene), to shift participant motivation to observe a scene or a face. After this, participants were more likely to label ambiguous images as those with the reward, and this was the case regardless of whether their perceptions were incorrect. These results suggest that agentic motivations heavily influence what visual information is attended to, but that agentic beliefs can remain rigid even when contradictory evidence is shown.

What accounts for this inability to update priors even when clear contradictory information is available? Active inference presumes that social agents do not come into any social perceptual process as passive perceivers of information. Motivations, context, and intentions can bias which priors are being acted upon and tested during the perceptual process (i.e., what information is sought out and thus attended to (Otten et al., 2017)). Put simply, prior precision influences what is being attended to. Moreover, what is being attended to is more likely to solicit a stronger prediction error signal when there is a mismatch between prediction and sense data (Friston, 2010). However, when attention toward a stimulus increases, error signals will be given more weight, and when attention is decreased, error signals are given less weight (Friston, 2010; Hung, 2023). This means that prediction errors not being attended to during perception will be ascribed less precision and thus be less likely to influence the posterior belief. In terms of group-based priors, this means that counter-stereotypical information is down-weighted during the perceptual process due to the increased attention on stereotype-consistent information—particularly for stronger (i.e., more precise) prejudicial priors (Hung, 2023). In a complementary fashion, the relative imprecision of sensory evidence (e.g., ambiguous stimuli) also allows priors to dominate. This follows because the relative contribution of the prior and (likelihood) of sensory evidence rests upon their relative precision. In the predictive processing literature, Bayesian belief updating is often described as minimizing precision-weighted prediction error (Hohwy, 2012). This is important because the precision *per se* has to be predicted, leading to a formal notion of attention as affording a high degree of confidence or precision to various sources of evidence (i.e., prediction errors). The posterior belief is therefore more likely to fall in line with the initial group-based prior (i.e., stereotype-consistent information). Over time, this may be what renders group-based priors and stereotypes difficult to undo.

In summary, we suggest some strengths of predictive processing, which are as follows: (1) its ability to explain biased perceptions in conditions of uncertainty, (2) it can explain how people arrive at biased perceptions of ambiguous stimuli, (3) it explains well how contextual conditions push perception towards biased perceptions, and (4) it provides a comprehensive account of why the most prejudiced people would be the least likely to revise biased beliefs. Below, we outline future directions and predictions that could test the predictive processing account of prejudice.

## Future directions

We have thus far outlined evidence for top-down effects of motivation, environment, and strength of priors for biased social processing and outlined why predictive processing may be a robust framework for explaining such findings. Below, we discuss future research avenues that could test tenets of predictive processing in intergroup perception.

### Perceptual versus post-perceptual effects

The studies reviewed here show that people *report* seeing different emotions or objects or respond differently to different faces or objects, depending on their existing knowledge about social groups and individuals. This suggests that stereotypes and prejudice that priors shape initial precepts in the earliest stages of intergroup perception, and more so when sense data is ambiguous (see above). Nonetheless, the observed effects of stereotypes could rely on changes in how the participants respond to these precepts.

To establish whether behavioral response patterns indicating the influence of group-based priors are the result of perceptual or response-related effects, reaction time and accuracy data could be analyzed using methods that can distinguish between response biases and perceptual effects. Signal detection theory (Stanislaw and Todorov, 1999) analyses the patterns of responding (hits, misses, correct rejections, and false alarms) to determine how easily two categories of stimuli can be perceptually distinguished from each other (d’) and how far the responders are biased to respond with one of the two response options (the criterion). For example, if the presence of a Black face prime makes it easier to perceptually distinguish guns from tools, then this should be reflected in a shift in d’ compared to white face primes (see, for example, Greenwald et al., 2003). However, if participants are more likely to respond “gun” after seeing a Black face compared to a white face, this will only shift the criterion (Payne et al., 2005; Klauer and Voss, 2008). Some research suggests that, at least with respect to the gun/tool task, there are no perceptual effects (Klauer and Voss, 2008; Burke, 2015; Payne and Correll, 2020). Future research, however, could apply this paradigm to face and emotion perception. For example, to see whether the presence of a black face makes one more likely to perceive anger, and vice versa.

Drift diffusion modeling (Ratcliff and McKoon, 2008) considers patterns of responding related to reaction times to estimate perceptual processing and response biases. From the resulting parameters, the drift rates and the starting point are particularly relevant to distinguish perceptual effects from response bias. On the one hand, the drift rate indicates how quickly sensory evidence is collected toward a specific response (say, identifying a face as angry). On the other hand, the starting point shows whether the system is already biased toward one response option (angry) over the other (happy) before any sensory information is processed. Again, this could be employed to see whether a prejudicial starting point pushes people to learn an association more quickly between some negative feature or emotion for outgroup members. Because active inference provides a formal (mathematical) account of sentient responses, it is possible to model empirical choice behavior or responses under a suitable generative model. By adjusting the priors of this model, one can estimate the prior beliefs held by a subject by optimizing the priors in the generative model to maximize the likelihood of a subject’s responses. This is known as computational phenotyping (Schwartenbeck and Friston, 2016) and has been used to phenotype psychiatric cohorts and neurotypical subjects in a variety of paradigms (Smith et al., 2019, 2020).

Future neuroscientific could also provide insights into the perceptual effects of group-based priors. Electrophysiology, with its high temporal resolution, can illuminate whether group-based priors alter early perceptual or later evaluation and response-related processing. An example of this approach could be seen whether people are more likely and quicker to collect evidence for anger in a Black face than in a White face. If people are put into a fearful or anger-inducing context, it could be that a Black face could be more likely to be inferred from an ambiguous face, an effect which may be modulated by an enhanced alpha response (Mayer et al., 2015).

Another way to employ neuroscience to pinpoint perceptual effects is through decoding the representations of sensory stimuli in the brain. Multivoxel pattern analysis (MVPA; Haxby et al., 2014) identifies patterns of activation related to specific categories of stimuli, for example, distinguishing between the patterns of brain activity related to angry and happy faces. With regard to the effects of group-based priors on social perception, classifiers could be employed to test whether the sensory representation of prior-consistent sensory input is sharpened (Kok et al., 2014) or whether established categories (for example, the neural patterns associated with angry or happy facial expressions) generalize to stereotype-induced perceptual categories [for example, ambiguous white and Black faces that appear more or less angry based on the race of the face (Hugenberg and Bodenhausen, 2003)].

### Testing prior probabilities

In predictive processing, if the top-down signal can account for the bottom-up prediction error, the prediction error is resolved or ‘explained away’ by inhibitory synaptic mechanisms (Bastos et al., 2012). If the prediction error cannot be explained away, it propagates the hierarchy to update posterior beliefs. These updates then furnish descending predictions that resolve prediction errors until they are explained away—and there is no further drive for updating. Alternatively, if the error signal cannot be explained, it propagates all the way up the perceptual hierarchy, and the internal generative model is updated to account for this error. This error can also be resolved by active inference (see above). In more formal terms, this process is described as Bayesian belief updating, where message passing between neurons consists of an ongoing reciprocity of matching top-down predictions with bottom-up prediction errors (Friston et al., 2017). This ongoing exchange of cascading neural signaling throughout the cortex is therefore the process of building—i.e., inverting and learning—a hierarchically generative model of the world. This process allows the cognitive system to update what it ‘expects’ to sample during perception and to update its model so that it can minimize prediction errors in the future (Friston, 2010).

This tenant of hierarchical predictive processing also makes it easy to see how stereotypes can be used as explanations for otherwise ambiguous sensory data in intergroup perception. As such, what follows from this framework is that only when encountering information highly contradictory to group-based priors do perceptually implemented stereotypes/prejudices become amendable to update. Higher-order beliefs drive the process of perception and belief generation. As such, it is not surprising that (depending on an agent’s motivations and attentional preferences discussed above) higher-order priors may not shift and change as rapidly as those lower on the hierarchy (Kiebel et al., 2008; Eil and Rao, 2011; Huntenburg et al., 2018). For example, when people are asked to guess the probability they will get cancer, they tend to ignore information that goes against their existing belief structures (Sharot et al., 2011). This shows that if information goes against higher order beliefs, and run contrary to an agent’s motivations or desires, they are less likely to be updated. The crucial point, then, is that not only does this predictive process occur in a hierarchical manner, but that higher order priors can be less amendable to update if the existing higher order predictions are positive for the agent and the incoming evidence is negative for the agent. This could be tested further in experimental settings. Researchers could alter prior probabilities of stereotypes to groups and then implement reversal learning paradigms to see how the brain updates the stereotype-group link under varying degrees of prior invariance (see Table 1 for more suggestions). This would allow researchers to assess the degree to which the brain updates the stereotype-group link and why it may or may not do Bayes optimally.

## Summary and conclusion

Here, we proposed that predictive processing can offer a framework to explain how prejudiced perception unfolds, develops, and maintains itself over time and why prejudiced priors are difficult to undo. Here, we outlined how biased priors can prejudice early intergroup perceptual processes. We then outlined how these findings fit within a predictive processing framework, applying this framework to understanding intergroup perception and cognition, and highlighted its explanatory power when compared with other prominent theories. We then outlined how future work could test the predictive processing hypothesis in the context of intergroup perception, offering specific hypotheses. While intergroup neuroscience is still somewhat new (Amodio, 2014; Amodio and Cikara, 2021), we contend that the adoption of novel frameworks such as predictive processing will offer substantive contributions to the area of intergroup perception, providing novel avenues forward for continued theoretical and empirical advancement.

## Data availability statement

The original contributions presented in the study are included in the article/supplementary material, further inquiries can be directed to the corresponding author.

## Author contributions

HM: Conceptualization, Writing – original draft, Writing – review & editing. MO: Conceptualization, Supervision, Writing – original draft, Writing – review & editing.
